# TIM-1 defines a human regulatory B cell population that is altered in frequency and function in systemic sclerosis patients

**DOI:** 10.1186/s13075-016-1213-9

**Published:** 2017-01-19

**Authors:** Octavio Aravena, Ashley Ferrier, Madhvi Menon, Claudia Mauri, Juan Carlos Aguillón, Lilian Soto, Diego Catalán

**Affiliations:** 1 Programa Disciplinario de Inmunología, Instituto de Ciencias Biomédicas (ICBM), Facultad de Medicina, Universidad de Chile, and Millennium Institute in Immunology and Immunotherapy, Santiago, Chile; 2 Centre for Rheumatology Research, Department of Medicine, University College London, London, UK; 3 Departamento de Medicina, Hospital Clínico, Universidad de Chile, Santiago, Chile

**Keywords:** Regulatory B cells, TIM-1, Systemic sclerosis, IL-10

## Abstract

**Background:**

Systemic sclerosis (SSc) is a systemic autoimmune disease characterized by excessive production of extracellular matrix by fibroblasts on skin and internal organs. Although Th2 cells have been involved in fibroblast stimulation, hyperactivated B cells may also play an important role. Regulatory B cells (Bregs) are cells capable of inhibiting inflammatory responses and controlling autoimmune diseases. Although many Breg populations have in common the ability to produce high amounts of IL-10, a unique surface marker defining most human Bregs is lacking. It has been described in mice that T cell Ig and mucin domain protein 1 (TIM-1) is an inclusive marker for Bregs, and that TIM-1+ B cells are able to prevent the development of autoimmunity. The aim of this work was to evaluate TIM-1 as a marker for human IL-10^+^ Bregs, and to determine whether TIM-1+ B cells are defective in SSc patients.

**Methods:**

SSc patients (n = 39) and 53 healthy subjects were recruited. TIM-1 and IL-10 expression was assessed in resting or activated peripheral blood CD19^+^ B cells by flow cytometry. The regulatory function of TIM-1^+^ or activated B cells from SSc patients and healthy subjects was assessed in autologous and allogenic co-cultures with CD4^+^ T cells, where T cell proliferation and IFN-γ, IL-17, TNF-α and IL-4 production by T cells was measured by flow cytometry.

**Results:**

TIM-1 and IL-10 were preferentially expressed in transitional B cells, but were upregulated in naïve and memory B cells upon stimulation. The frequency of transitional TIM-1^+^ IL-10^+^ B cells was significantly decreased in SSc patients compared to healthy controls. In addition, activated B cells from SSc patients induced stronger allogenic Th1 and Th2 responses than activated B cells from healthy controls. Finally, TIM-1^+^ B cells, including transitional and non-transitional cells, exhibited a higher CD4^+^ T cell suppressive ability than TIM-1^−^ B cells in healthy controls, but not in SSc patients.

**Conclusions:**

TIM-1 is a unique marker for the identification of a human IL-10^+^ Breg subpopulation which is partially superimposed with transitional B cells. Alterations in TIM-1^+^ B cells could contribute to the development of autoimmune diseases such as SSc.

**Electronic supplementary material:**

The online version of this article (doi:10.1186/s13075-016-1213-9) contains supplementary material, which is available to authorized users.

## Background

B cells play a central role in immune homeostasis, not only as precursors of antibody-secreting plasma cells, but also by presenting antigens and activating T cells, secreting a multiplicity of cytokines, and performing immune regulatory functions [1, 2]. The maintenance of immune tolerance and prevention of autoimmunity is exerted by different subpopulations of regulatory B cells (Bregs), which include type 2 marginal zone precursors [3], CD1d^high^CD5^+^ B10 cells [4], plasmablasts [5], and plasma cells [6] in mice; and CD24^high^CD38^high^ transitional B cells [7], CD24^high^CD27^+^ B10 cells [8], and CD25^high^CD86^high^ B cells [9] in humans. Nearly all of them have been functionally classified as regulatory based on their ability to secrete interleukin (IL)-10, suppress the differentiation or activation of pro-inflammatory immune cells, such as monocytes, dendritic cells, CD4^+^ T cells, and cytotoxic CD8^+^ T cells and/or induce the differentiation or activation of regulatory T cells and invariant natural killer T (iNKT) [10]. However, a unique marker common to most human Breg populations has not been found so far.

Studies in mice have postulated T cell Ig and mucin domain protein 1 (TIM-1) as an inclusive marker for IL-10^+^ Bregs [11–13]. TIM-1 binds to phosphatidylserine, which is flipped to the outer leaflet of apoptotic cell membranes, conveying phagocytosis by macrophages and IL-10 expression by B cells [11, 14]. Adoptive transfer of TIM-1^+^ B cells prevents allograft rejection and attenuates the development of experimental autoimmune encephalomyelitis (EAE) [12], while susceptible mice with a mutated TIM-1 molecule develop accelerated lupus [13]. Although TIM-1^+^ cells have been found to be enriched in IL-10-expressing human B cells [15–17], their regulatory function and their association with systemic autoimmune diseases have been insufficiently characterized.

Systemic Sclerosis (SSc) is a systemic autoimmune disease with pathophysiological features based on three phenomena: autoimmunity, fibrosis and vasculopathy, which in conjunction lead to a complex pattern of manifestations that include an excessive deposition of extracellular matrix on skin and internal organs, transient vasoconstriction events, and the production of a wide spectrum of autoantibodies [18]. This disease is classified in limited cutaneous (lcSSc) and diffuse cutaneous (dcSSc), according to the degree of skin sclerosis, internal organ involvement, and autoantibody profile [18].

Patients with SSc have a high frequency of circulating and skin-infiltrating type 2 CD4^+^ T helper cells (Th2) producing profibrotic cytokines such as IL-4 [19, 20]. More recently, IL-17 and IL-22-producing CD4^+^ T cells (Th17 and Th22, respectively) were also found to be expanded in blood from patients with SSc and have been related to SSc pathogenesis [21, 22]. Furthermore, regulatory cells, such as CD4^+^ regulatory T cells (Tregs), that keep those pathogenic populations in check, are defective in patients with SSc [23].

B cells exhibit a hyperactivated phenotype in patients with SSc, with high expression of activation molecules and inflammatory cytokines [24, 25], but low expression of IL-10 [26–28]. Moreover, several reports have confirmed the benefits of B cell depletion therapies on skin fibrosis and lung function in patients with SSc [29]. However, it is not known whether Bregs from patients with SSc are able to restrain the activation of pro-inflammatory CD4^+^ T cell responses.

Transitional B cells have been previously ascribed with regulatory functions; however, only around 15% of them produce IL-10 [7]. Therefore, we set out to investigate whether TIM-1 could better identify the IL-10-producing population amongst transitional B cells. In addition, we investigated the presence of functional alterations in TIM-1-expressing B cells in Th2-driven systemic autoimmune disease with hyperactivated B cells such as SSc. Results herein show that TIM-1 identifies most IL-10^+^ B cells amongst transitional B cells. We also show that the frequency of TIM-1^+^ transitional B cells, but not of other B cell subsets, was reduced in patients with SSc compared to healthy controls. In addition, we observed that activated B cells from patients with SSc potentiate Th1 and Th2 responses, instead of suppressing CD4^+^ T cell responses as in healthy donors. Finally, while transitional and non-transitional TIM-1^+^ B cells from healthy subjects suppressed CD4^+^ T cell activation, TIM-1^+^ B cells from patients with SSc did not, suggesting a functional defect of Bregs in this disease.

## Methods

### Patients and controls

Peripheral blood samples from 39 patients with SSc meeting the American College of Rheumatology/European League Against Rheumatism Classification Criteria for SSc [30], and 53 healthy controls, were obtained for B cell characterization, and purification of B and T cells. Characteristics of SSc and healthy controls can be found in Table 1. This study was approved by the Ethical Committees of the Hospital Clínico and Facultad de Medicina, Universidad de Chile, and UCLH-National Health Service Trust, London, UK. All subjects gave written informed consent in accordance with the Declaration of Helsinki.

**Table 1:** Main demographic and clinical characteristics of the patients with systemic sclerosis and healthy controls

Characteristics	Patients (n = 39)	Controls (n = 53)
Female/male, *n*	30/9	28/25
Age, mean ± SD	48.4 ± 11.3	40.0 ± 13.7
Disease duration, months, mean ± SD	95.4 ± 100.7	
lcSSc/dcSSc, *n*	26/13	
Rodnan score, mean ± SD	13.8 ± 6.8	
Corrected DLCO^a^, mean ± SD	19.7 ± 6.7	
ANA pattern^b^, *n* (%)		
Speckled	10/34 (29.4)	
Nucleolar	9/34 (26.5)	
Homogeneous	9/34 (26.5)	
Centromere	16/34 (47.1)	
Anti-Scl-70 positivity	6/33 (18.2)	
Organ involvement, *n* (%)		
Peripheral vascular	16 (41.6)	
Gastrointestinal tract	27 (69.2)	
Lung	21 (53.8)	
Heart	16 (41.0)	
Kidney	4 (10.2)	
Therapy, *n*/total number		
Prednisone	3/39	
Azathioprine + prednisone	2/39	
Methotrexate	4/39	
D-penicillamine	1/39	
Methotrexate + D-penicillamine	1/39	
Methotrexate + D-penicillamine + prednisone	1/39	
Hydroxychloroquine	4/39	
Methotrexate + hydroxychloroquine	1/39	
Only symptomatic treatment	22/39	

^a^Measured in 25 patients. ^b^Some patients have more than one pattern. *SD* standard deviation, *lcSSc* limited cutaneous systemic sclerosis, *dcSSc* diffuse cutaneous systemic sclerosis, *Corrected DLCO* Diffusing capacity for carbon monoxide correction of predicted value for hemoglobin, *ANA* antinuclear antibody

### Flow cytometry and cell sorting

Dead cells were excluded from flow cytometry analysis and cell sorting using the LIVE/DEAD® staining kit (Thermo Fisher Scientific, Waltham, MA, USA). The following anti-human antibodies were used for flow cytometry or cell sorting: anti-CD19 FITC (clone HIB19), anti-CD24 PECy7 (clone ML5), anti-CD38 APC (clone HB-7), anti-IL-10 PE (clone JES3-9D7), anti-TIM-1 PE (clone 1D12), anti-CD3 APC (clone SK7), anti-interferon (IFN)-γ (clone 4S.B3), anti-IL-4 PE (clone MP4-25D2), and anti-IL-17 PerCP (clone BL168) (BioLegend, San Diego, CA, USA). To assess co-expression of IL-10 and TIM-1 on B cells, an anti-TIM-1 Alexa Fluor 488 antibody (clone 219211; R&D Systems Inc, MN, USA) was used. Intracellular cytokines were stained using Permeabilization and IC Fixation Buffers (eBioscience, San Diego, CA, USA). Samples were acquired and sorted with a FACSAria III cell sorter (Becton Dickinson, NJ, USA), and data was analyzed with the FloJo X Software (OR, USA).

### B and T cell isolation

Untouched CD19^+^ B cells and CD4^+^ T cells were isolated from fresh heparinized whole blood or buffy coats with the RosetteSep Human B cell or CD4^+^ T Cell Enrichment Cocktail kits, respectively (Stemcell Technologies, Vancouver, Canada).

### B cell activation

Isolated B cells were cultured for 48 hours in RPMI 1640 medium supplemented with 10% fetal bovine serum (HyClone, GE Healthcare, USA) at a 1 × 10^6^ cells/ml density, 37 °C and 5% CO_2_, in presence or absence of 5 μg/ml polyclonal anti-human IgG + IgM goat antibodies (Jackson Immunoresearch, West Grove, PA, USA) to activate the B cell receptor (BCR) and 3 μg/ml ODN 2006 Class B CpG oligonucleotide to activate Toll-like receptor 9 (TLR9) (Invivogen, San Diego, CA, USA). To evaluate IL-10 secretion by ELISA (Biolegend), cells were stimulated with 50 ng/ml phorbol 12-myristate 13-acetate (PMA) and 1 μg/ml ionomycin (Sigma-Aldrich, Saint Louis, MO, USA) for the last 5 hours of culture, and for intracellular detection of cytokines by flow cytometry, 1 μg/ml brefeldin A (eBioscience) was simultaneously added.

### CD4^+^ T cell and B cell co-cultures

For autologous co-cultures, total B cells and CD4^+^ T cells from healthy donors were isolated. B cells were cultured for 48 hours in absence of stimulus or were stimulated with anti-BCR antibodies plus CpG, as indicated above. Next, B cells were harvested, washed thoroughly and plated at a 1:1 ratio with 5,6-carboxylfluorescein diacetate succinimidyl ester (CFSE)-labeled autologous CD4^+^ T cells (1 × 10^6^ total cells/ml) and anti-CD3/anti-CD28 antibody-conjugated beads (Life Technologies, Paisley, UK) for 5 days. To assess the regulatory ability of activated B cells from patients with SSc, an allogenic assay was performed. B cells from patients with SSc or sex-matched and age-matched healthy donors, either unstimulated or stimulated for 48 hours, were co-cultured for 4 days at a 1:1 ratio with CFSE-labeled CD4^+^ T cells from a single healthy donor, in order to exclude the intrinsic cytokine profile of CD4^+^ T cells from patients with SSc [21]. In some cases, TIM-1^+^ and TIM-1^−^ CD19^+^ B cells from patients with SSc or healthy donors were sorted by fluorescence-activated cell sorting, and immediately plated with autologous CFSE-labelled CD4^+^ T cells at a 1:1 ratio and anti-CD3/anti-CD28 beads for 5 days. In another experiment, TIM-1^+^ CD24^high^ CD38^high^ transitional B cells, TIM-1^−^ CD24^high^ CD38^high^ transitional B cells and TIM-1^+^ CD24^med/low^ CD38^med/low^ non-transitional B cells from healthy donors were isolated by cell sorting and were cultured with autologous CD4^+^ CD25^−^ T cells stimulated with 0.5 ug/ml plate-bound anti-CD3 antibody, at a 1:2 ratio for 3 days. For all co-cultures, 50 ng/ml PMA, 1 μg/ml ionomycin and 1 μg/ml brefeldin A was added for the last 5 hours, and intracellular IFN-γ, IL-4, IL-17 or TNF-α expression in CD4^+^ T cells was determined by flow cytometry. An inhibition index was calculated according to the following formula:

Inhibition index = 1 - (Percentage of cytokine-producing CD4+ T cells in presence of B cells/Percentage of cytokine-producing CD4+ T cells activated with anti-CD3/anti-CD28 alone).

### Statistical analyses

The two-tailed unpaired or paired Student *t* test was used when appropriate to make comparisons between two conditions or between patients with SSc and healthy controls. Analysis of variance (ANOVA) for repeated measures with Bonferroni post-test correction was used for comparisons between B cell subpopulations. The Spearman test was used to test for correlation between continuous variables. A *P* value <0.05was considered significant. All analyses were performed with GraphPad Prism 6 (La Jolla, CA, USA).

## Results

### Patients with SSc exhibit reduced peripheral blood TIM-1+ transitional B cells

**Fig. 1:**
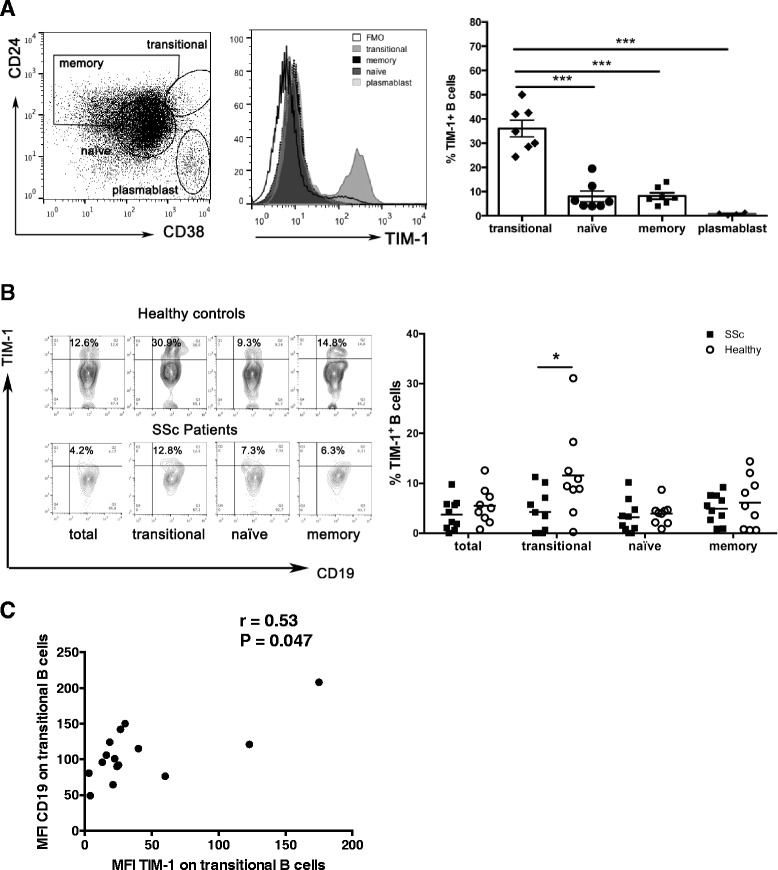
T cell Ig and mucin domain protein 1 (*TIM-1*) expression by healthy and systemic sclerosis (*SSc*) B cell subpopulations. **a** *Left* representative dot-plot indicating the gating strategy to identify transitional, naïve, memory, and plasmablast B cell subpopulations according to the expression of CD24 and CD38. *Middle* representative histograms show the expression of TIM-1 in different B cell subpopulations. *Right* percentage of TIM-1-expressing B cells from healthy donors in each indicated subpopulation, n = 7. **b**. Representative dot-plots (*left*) and graph (*right*) show the frequency of TIM-1^+^ cells on different B cell subpopulations from healthy subjects and patients with SSc. **c** Correlation between the expression levels of TIM-1 and CD19 in transitional B cells from patients with SSc. *FMO* fluorescence minus one staining control, *MFI* mean fluorescence intensity. *P < 0.05, ***P < 0.001

First, the expression of TIM-1 was evaluated on different human circulating B cell subpopulations. For this purpose, peripheral blood mononuclear cells were isolated and CD19^+^ B cell subpopulations were defined by flow cytometry according to the expression of CD24 and CD38 in: CD19^+^ CD24^high^ CD38^high^ transitional B cells, CD19^+^ CD24^med^ CD38^med^ naïve B cells, CD19^+^ CD24^high^ CD38^med^ memory B cells, and CD19^+^ CD24^−^ CD38^high^ plasmablasts, as described elsewhere [31] (Fig. 1a). As shown in Fig. 1a, TIM-1 was expressed in all subpopulations except plasmablasts. Transitional B cells exhibited a markedly higher frequency of TIM-1-expressing cells (around 35%), compared to naïve and memory cells (Fig. 1a).

Defects in different subsets of Bregs have been described in several autoimmune diseases [7, 32]. Our group has previously reported that compared to healthy controls, patients with SSc, have an increased frequency of transitional B cells, but a decreased proportion of IL-10-producing transitional B cells after short stimulation with PMA and ionomycin, and also a decreased frequency of CD25^high^ CD27^high^ CD86^high^ CD1d^high^ B cells, which are regarded as regulatory [9, 28]. To assess whether defects in the expression of TIM-1 in B cells are associated with the development of SSc, frequencies of TIM-1^+^ B cells in peripheral blood of patients with SSc and healthy controls were compared. Reduced frequency of B cells expressing TIM-1 was observed exclusively in the transitional subpopulation in patients with SSc (Fig. 1b).

The hyperactivated phenotype of B cells in SSc has been attributed to exacerbated activity of the activating molecule CD19, and CD19 expression levels have been found to be increased in naïve and memory B cell subpopulations in patients with SSc [24, 33, 34]. Recently, we have demonstrated that CD19, together with activation markers CD86 and CD40, are upregulated in transitional B cells from patients with SSc [28]. Correlation analysis was performed to find out if there is a relationship between reduced expression of TIM-1 and the hyperactivated phenotype of SSc B cells. Although there was no significant correlation between the expression of TIM-1 and CD86 or CD40 (data not shown), there was direct correlation between the expression of TIM-1 and CD19 in transitional B cells (Fig. 1c).

### BCR and TLR9 activation induces an increase in TIM-1 expression that is impaired in B cells from patients with SSc

**Fig. 2:**
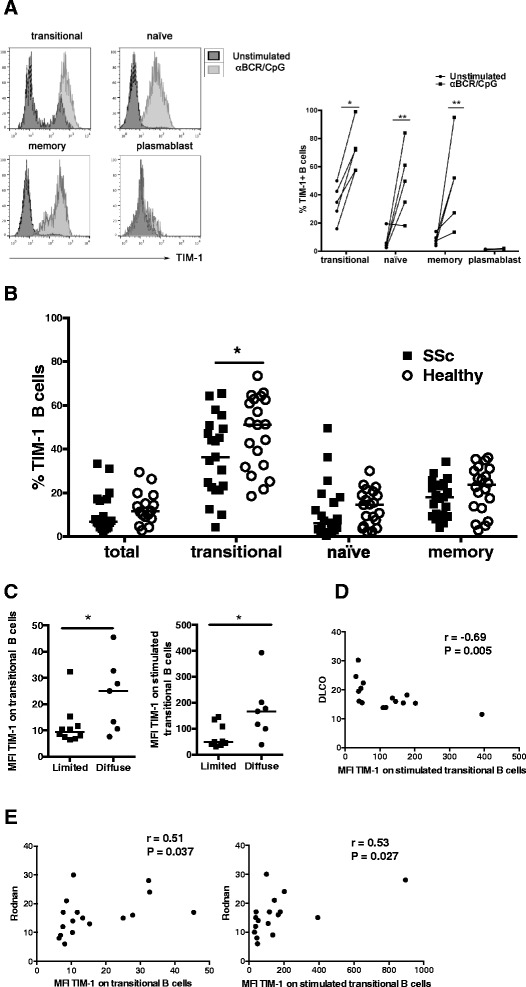
T cell Ig and mucin domain protein 1 (*TIM-1*) expression upon B cell receptor (BCR) and Toll-like receptor-9 (TLR9) activation in healthy subjects and patients with systemic sclerosis (*SSc*). **a. a** Representative histograms (*left*) and summarizing graph (*right*) show the percentage of TIM-1-expressing B cells from healthy subjects in different subpopulations, left unstimulated or activated with an anti-BCR antibody (*αBCR*) and CpG for 48 hours. **b** Percentage of TIM-1^+^ total, transitional, naïve and memory B cells from healthy controls and patients with SSc after stimulation with αBCR and CpG for 48 hours. **c** Expression levels of TIM-1 on unstimulated (*left*) or stimulated (*right*) transitional B cells from patients with SSc with the diffuse or limited form of the disease. **d** Correlation between the expression levels of TIM-1 in stimulated transitional B cells from patients with SSc and the diffusing capacity for carbon monoxide (DLCO). **e**. Correlation between the expression levels of TIM-1 in unstimulated (*left*) or stimulated (*right*) transitional B cells from patients with SSc and the Rodnan score of skin involvement. *MFI* mean fluorescence intensity. *P < 0.05, **P < 0.01

Combined stimulation of the BCR and TLR9 in human B cells induces robust IL-10 secretion and equips them with the ability to suppress T cell activation [35]. Therefore, the possibility that TIM-1, as a marker of Bregs, could change its expression after B cell activation was explored. A significant increase in TIM-1 expression upon activation of BCR and TLR9 was observed in all studied B cell subpopulations except plasmablasts (Fig. 2a). Similar to constitutive TIM-1 expression on transitional B cells, the percentage of transitional B cells expressing TIM-1 after activation of BCR and TLR9 was lower in patients with SSc than in healthy controls (Fig. 2b).

Activation of B cells has been involved with lung and skin fibrosis in a murine model of SSc [36]. Accordingly, lungs from SSc-associated interstitial lung disease and skin samples from patients with SSc exhibit B cell infiltration [37, 38]. Moreover, B cell depletion therapy has shown a beneficial effect on lung function and skin fibrosis in patients with SSc [39]. Therefore, we assessed a possible association between TIM-1 expression and parameters related to lung and skin fibrosis. Interestingly, TIM-1 expression levels on transitional B cells, either unstimulated or stimulated, were higher in patients with SSc who presented with the diffuse cutaneous form of the disease (Fig. 2c). Moreover, there was correlation between worse respiratory function, measured as a lower DLCO, and higher TIM-1 expression levels on stimulated transitional B cells (Fig. 2d). Likewise, there was direct correlation between TIM-1 levels on unstimulated or stimulated transitional B cells and the Rodnan score, which mirrors the degree of skin fibrosis (Fig. 2e). There was no significant association between TIM-1 expression on B cells and the presence of autoantibodies or involvement of internal organs.

### A subpopulation of human transitional B cells co-express TIM-1 and IL-10, an ability that is compromised in B cells from patients with SSc

**Fig. 3:**
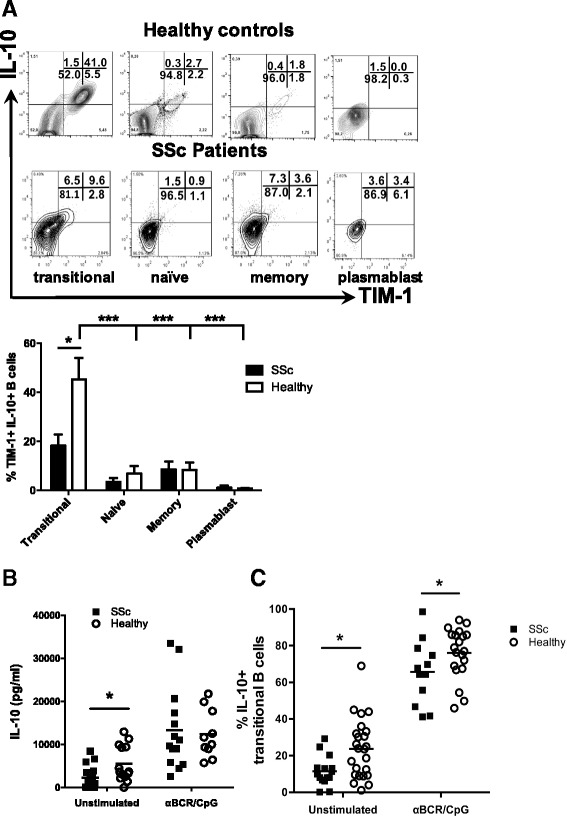
Co-expression of IL-10 and T cell Ig and mucin domain protein 1 (*TIM-1*) in B cell subpopulations from healthy subjects and patients with systemic sclerosis (*SSc*). **a** Representative dot-plots (*above*) and column graph (*below*) show the percentage of TIM-1 and IL-10 expression on different B cell subpopulations from healthy controls and patients with SSc stimulated with phorbol 12-myristate 13-acetate (PMA) and ionomycin for 5 hours (n = 3 patients with SSc, n = 5 healthy controls). **b** Levels of IL-10 secreted by total B cells from healthy controls and patients with SSc stimulated with PMA and ionomycin alone or after incubation with an anti-B-cell receptor antibody (*αBCR*) and CpG for 48 hours. **c**. Percentage of IL-10^+^ transitional B cells from healthy controls and patients with SSc stimulated under the same conditions as in **b**. *P < 0.05, ***P < 0.001

So far, the main common trait for all human regulatory B cell populations is the expression of IL-10. In order to verify whether human TIM-1 is preferentially expressed on IL-10-producing B cells, as previously shown in mice [14], B cells were isolated and stimulated with PMA and ionomycin, and co-expression of surface TIM-1 and intracellular IL-10 was assessed by flow cytometry. As previously described [7], transitional B cells were the subpopulation most enriched in IL-10^+^ cells (Fig. 3a, upper panel). More importantly, TIM-1 was expressed in the majority of transitional IL-10^+^ B cells, while naïve, memor, and plasmablast TIM-1^+^ B cells did not show major IL-10 expression (Fig. 3a, upper panel). Furthermore, about 60% of transitional B cells, but also around 30% of memory B cells, co-expressed IL-10 and TIM-1 after activation with CpG and anti-BCR (Additional file 1: Figure S1). In contrast, patients with SSc had significantly decreased TIM-1^+^ IL-10^+^ transitional B cell frequencies after 5 hours of incubation with PMA and ionomycin (Fig. 3a, lower panel and graph). Thus, similar to murine B cells, TIM-1 was preferentially expressed in IL-10-producing B cells from healthy donors, but not from patients with SSc. These differences are in line with decreased IL-10 secretion by B cells stimulated with PMA/ionomycin, and a reduced frequency of IL-10-producing transitional B cells, either stimulated with PMA/ionomycin alone or pre-activated with an anti-BCR antibody and CpG, observed in patients with SSc (Fig. 3b-c).

### B cells from patients with SSc activated via BCR and TLR9 are unable to inhibit allogenic CD4^+^ T cell responses

**Fig. 4:**
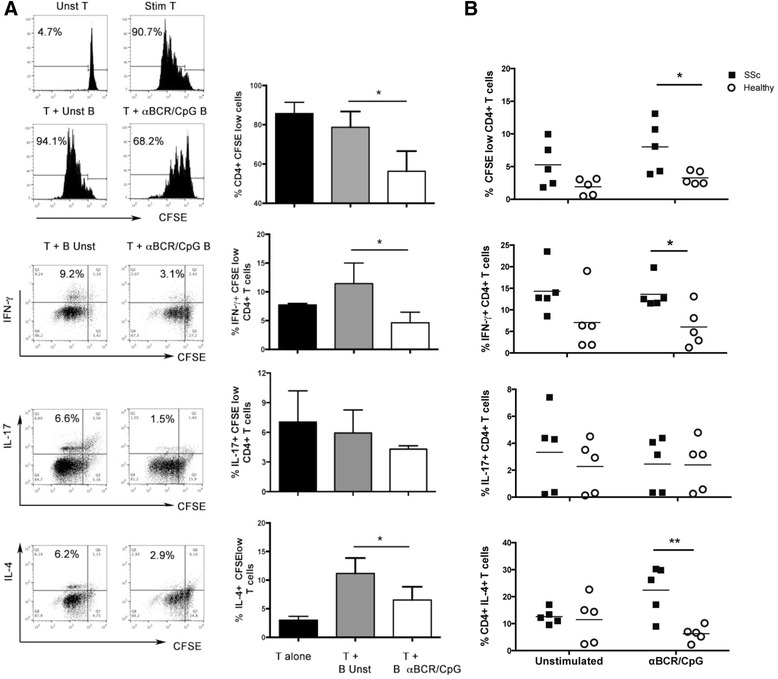
Modulation of CD4^+^ T cells by stimulated B cells from healthy controls and patients systemic sclerosis (*SSc*). **a** Representative histogram and dot-plots (*left*), and graphs (*righ*t) showing the effect of B cells from a healthy control stimulated with an anti-B-cell receptor antibody (*αBCR*) and CpG for 48 hours, in the proliferation (measured as percentage of 5, 6-carboxylfluorescein diacetate succinimidyl ester (*CFSE*)^low^ cells) (n = 5) and in the production of interferon (*IFN*)-γ (n = 7), IL-17 (n = 4) and IL-4 (n = 6) by autologous CD4^+^ T cells stimulated with anti-CD3 and anti-CD28 antibodies for 5 days. CD4^+^ T cells cultured alone (*unst T*) or only with stimulating antibodies (*stim T*) were used as negative and positive controls, respectively. **b** Graphs represent the percentage of allogenic CD4^+^ T cells proliferating and producing IFN-γ, IL-17, and IL-4 after 4-day co-culture with B cells from patients with SSc and healthy controls, stimulated with αBCR and CpG, or left unstimulated for 48 hours. *P < 0.05, **P < 0.01

B cells activated either with CpG plus anti-BCR, or with anti-BCR alone, have been demonstrated to suppress CD4+ T cell proliferation [40, 41]. Thus, the effect of activated B cells expressing high levels of TIM-1 and IL-10 after stimulation with anti-BCR plus CpG, over polyclonally activated autologous CD4^+^ T cells was evaluated. Significantly lower T cell proliferation and IFN-γ and IL-4 expression was observed in co-cultures with activated B cells compared to unstimulated B cells, while no differences were detected in IL-17 expression (Fig. 4a).

Thereafter, the regulatory ability of activated B cells was compared between patients with SSc and healthy controls. B cells from patients with SSc or healthy donors, either unstimulated or stimulated for 48 hours with anti-BCR plus CpG, were co-cultured with CD4^+^ T cells from a single third-party healthy donor. As shown in Fig. 4b, stimulated B cells from patients with SSc induced greater proliferation and production of IFN-γ and IL-4 by allogenic CD4+ T cells, compared to healthy controls (Fig. 4b). These results indicate that stimulatory conditions that induce B cells to upregulate TIM-1 and IL-10, and endow them with regulatory functions, generate a Th1 and Th2 activation profile in B cells from patients with SSc.

### TIM-1^+^ B cells from healthy subjects, but not from patients with SSc, inhibit autologous CD4^+^ T cell responses

**Fig. 5:**
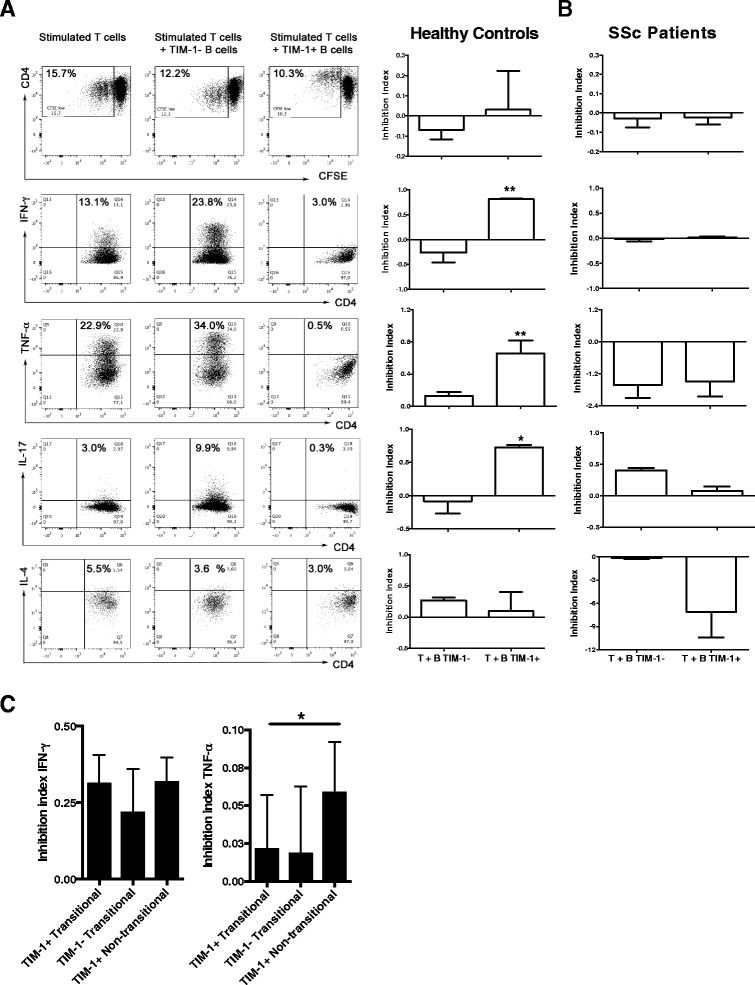
Suppressive function of T cell Ig and mucin domain protein 1 (*TIM-1*)^+^ B cells from healthy controls and patients with systemic sclerosis (*SSc*) over T cells. **a** *Left* representative dot-plots show the effect of TIM-1^+^ and TIM-1^−^ purified B cells from healthy controls in the proliferation (measured as percentage of 5, 6-carboxylfluorescein diacetate succinimidyl ester (*CFSE*)^low^ cells) and in the production of interferon (*IFN*)-γ, TNF-α, IL-17, and IL-4 by autologous CD4+ T cells stimulated with anti-CD3 and anti-CD28 antibodies for 5 days. *Right* suppressive effect of TIM-1^+^ and TIM-1^−^ B cells from healthy donors in the proliferation and the production of IFN-γ, TNF-α, IL-17, and IL-4 by autologous stimulated CD4^+^ T, calculated as the inhibition index (1 – (percentage of activated CD4^+^ T cells in presence of B cells/percentage of activated CD4^+^ T cells with anti-CD3 plus anti-CD28 alone)) (n = 3). **b** Suppressive effect of TIM-1^+^ and TIM-1^−^ B cells from patients with SSc in the proliferation, and the production of IFN-γ, TNF-α, IL-17, and IL-4 by autologous stimulated CD4^+^ T, calculated as inhibition index (n = 3). **c** Inhibition of the expression of IFN-γ and TNF-α by autologous activated CD4^+^ T cells by the indicated B cell subpopulations from healthy donors (n = 3). *P < 0.05, **P < 0.01

As TIM-1 defines an important population of IL-10-producing B cells, the regulatory properties of TIM-1^+^ B cells were evaluated. First, total TIM-1^+^ or TIM-1^−^ B cells from healthy subjects or patients with SSc were isolated by cell sorting. TIM-1^+^ or TIM-1^−^ B cells were co-cultured at a 1:1 ratio with autologous CD4^+^ T cells, which were stimulated with anti-CD3/anti-CD28 beads. No differences were observed in the proliferative response of CD4^+^ T cells co-cultured with TIM-1^+^ or TIM-1^−^ B cells from healthy controls; however, TIM-1^+^ B cells strongly suppressed the expression of pro-inflammatory cytokines by CD4^+^ T cells, such as IFN-γ, TNF-α, and IL-17, compared to TIM-1^−^ B cells (Fig. 5a). There was only modest inhibition of IL-4 production by CD4^+^ T cells with both TIM-1^+^ and TIM-1^−^ B cells (Fig. 5a). Conversely, TIM-1^+^ and TIM-1^−^ B cells from patients with SSc did not suppress CD4^+^ T cell proliferation or production of any of the cytokines assessed (Fig. 5b).

### Human TIM-1^+^ B cells and transitional B cells exhibit a different suppressive profile

As transitional B cells have been demonstrated to suppress T cell activation [7], and TIM-1 is mainly expressed on this subpopulation along with IL-10, an assay was performed to address whether TIM-1^+^ or transitional B cells, or both subpopulations, are responsible for the regulatory effect previously described for transitional B cells. Three B cell subpopulations were sorted: TIM-1^+^ CD24^high^ CD38^high^ (TIM-1^+^ transitional), TIM-1^−^ CD24^high^ CD38^high^ (TIM-1^−^ transitional), and TIM-1^+^ CD24^med/low^ CD38^med/low^ (TIM-1^+^ non-transitional). The sorted cells were co-cultured with autologous anti-CD3-activated CD4^+^ CD25^−^ T cells for 3 days, and the expression of IFN-γ and TNF-α was assessed by flow cytometry. As shown in Fig. 5c, although the three subpopulations suppressed the expression of IFN-γ, both TIM-1^+^ subpopulations tended to achieve it in a stronger way (Fig. 5c). In contrast, on evaluation of TNF-α suppression, even though the inhibition was modest and there was wide inter-individual variation, TIM-1^+^ non-transitional cells appeared to be more potent (Fig. 5c). These results suggest that TIM-1 identifies a population of human Bregs different from transitional B cells.

## Discussion

TIM-1 is an inclusive marker for IL-10^+^ Bregs and an important receptor for Breg induction and function in mice, probably by sensing of apoptotic cells and induction of IL-10 expression on B cells, in order to preserve tolerance to self-antigens and prevent autoimmunity [11, 12]. In humans, few studies have evaluated TIM-1 expression in B cells. Liu et al. report that TIM-1 is expressed in over 75% of peripheral IL-10^+^ B cells and less than 25% of IL-10^−^ B cells from healthy subjects [15]. In contrast, Kristensen et al. found that up to 40% of IL-10^+^ B cells express TIM-1, which is almost absent from IL-10^−^ B cells [16]. Similar to this observation, we show that among total B cells, around 50% of PMA/ionomycin-activated IL-10^+^ B cells are TIM-1^+^, whereas TIM-1 is expressed in only 10% of IL-10^−^ B cells. The different antibody clone used to stain TIM-1 in the earlier work could explain this divergence.

It has been shown that murine TIM-1^+^ B cells are able to suppress Th1 responses in vivo and promote Th2 and Treg responses in an allograft transplantation setting [12]. Also, TIM-1^+^ B cells inhibit Th1 and Th17 responses in vivo in the EAE model [13]. In humans, TIM-1 has been used as a surface marker to isolate Bregs and explore their in vitro suppressive function in HIV-infected patients, demonstrating an inhibition of antigen-specific IFN-γ and TNF-α production by CD8^+^ and CD4^+^ T cells [15]. Similarly, TIM-1^+^ B cells from patients with HBV-induced hepatocellular carcinoma do not suppress granzyme and perforin production by CD4^+^ T cells [17]. To determine whether TIM-1 identifies previously described regulatory B cell subpopulations, we evaluated TIM-1 expression in plasmablasts, transitional, naïve and memory B cells. Of interest, the transitional subpopulation, one of the better characterized human Breg subsets, was by far the most enriched in TIM-1^+^ cells, and the majority of TIM-1^+^ transitional B cells also co-expressed IL-10.

In addition, we found that TIM-1^+^ B cells from healthy donors have a potent suppressive capacity compared to their TIM-1^−^ counterparts, inhibiting the production of IFN-γ, TNF-α and IL-17 by activated CD4^+^ T cells. It is noteworthy that TIM-1^−^ transitional B cells are also able to suppress IFN-γ production by autologous CD4^+^ T cells, although not equivalently to TIM-1^+^ transitional B cells, and that non-transitional TIM-1^+^ B cells also suppress IFN-γ and TNF-α production, revealing that transitional B cells and TIM-1^+^ B cells probably correspond to two different, but partially superimposed, regulatory subpopulations. According to previous studies, IL-10 appears to be crucial in the inhibitory functions of TIM-1^+^ and transitional B cells [7, 15, 17]; however, the involvement of other mechanisms cannot be excluded.

After stimulation of BCR and TLR9 receptors, naïve and memory B cells acquired TIM-1 expression, together with upregulation of IL-10 production. Even upon stimulation, transitional B cells comprise the highest frequency of TIM-1^+^ and IL-10^+^ cells. Such TIM-1 induction upon BCR activation was previously demonstrated in murine germinal center B cells [42], and could be a possible explanation for the positive correlation we observed in transitional B cells from patients with SSc, between the expression levels of TIM-1 and CD19, a B cell activating co-receptor that has been previously reported to be upregulated in SSc B cells [28, 33]. These results could imply a general mechanism to favor IL-10 production by B cells in the context of an ongoing inflammatory response, where an accumulation of apoptotic cells carrying potential autoantigens and TLR ligands, bears the inherent risk of developing autoimmunity [43].

Until now, there have been only two studies published in which the frequency of TIM-1^+^ B cells has been evaluated in autoimmune disease [16, 44]. In the first, peripheral blood TIM-1^+^ IL-10^+^ B cells from patients with Graves’ disease and Hashimoto’s thyroiditis were found to be elevated compared to healthy donors [16]. In contrast, in patients with myasthenia gravis, the frequency of peripheral blood TIM-1^+^ B cells was lower than in healthy controls, and was negatively correlated with disease severity [44]. SSc is a systemic autoimmune disease with hyperactivated B cells having a prominent role in its pathogenicity [24, 25], and in consequence, it is a good model for the study of Breg frequency and function. According to our results, patients with SSc have reduced frequencies of TIM-1^+^ IL-10^+^ B cells, but only within the transitional subpopulation, both in resting cells and after stimulation of the BCR and TLR9 receptor. Differences between our study and the one in autoimmune thyroid disease may be due to the completely disparate pathogenic mechanisms behind organ-specific autoimmune diseases such as Graves’ disease and Hashimoto’s thyroiditis, and a systemic disease such as SSc. Additionally, this could also be due to the fact that no characterization of B cell subpopulations expressing TIM-1 was performed in that study.

We also found that TIM-1 expression levels on transitional B cells are higher in the diffuse form of the disease, and that they are directly correlated with parameters related to the degree of skin and lung fibrosis and inflammation, such as the Rodnan score and DLCO, respectively. These results are in line with our results showing upregulation of TIM-1 after TLR9 and BCR activation, and with evidence from mouse models showing increased frequency of Bregs in response to inflammation [10, 45].

Our results show that TIM-1^+^ B cells from patients with SSc are unable to suppress CD4^+^ T cell activation, and that stimulated B cells from patients with SSc induced stronger activation of Th1 and Th2 allogenic responses than those from healthy controls. Two studies have described reduced frequencies of IL-10-producing Bregs in patients with SSc, upon stimulation with CD40L and CpG [26], or CpG alone [27]. In the latter work, the authors described altered activation of STAT-3 and p38 MAPK, two signaling molecules involved in IL-10 production, after stimulation of the BCR and TLR9 receptor [27]. This evidence, together with our results, points to defective regulatory functions in Bregs from patients with SSc, which could be partially explained by their inability to increase TIM-1 and IL-10, and probably other inhibitory molecules, upon stimulation, while expressing activation molecules and pro-inflammatory cytokines, such as IL-6 [25, 28], tipping the balance toward a more pro-inflammatory or pro-fibrotic profile. Although it has been proposed that hyperactivated B cells directly or indirectly help CD4^+^ T cells to differentiate into a Th2 profile in SSc [29], this assumption had not been tested until now.

## Conclusions

Overall, we have demonstrated that TIM-1 is a viable marker for IL-10^+^ Bregs in humans and that TIM-1^+^ B cells are decreased in frequency and have an impaired regulatory function in patients with SSc. The results presented herein do not only contribute to the characterization of a novel marker for a subpopulation of B cells with regulatory properties, but also open new routes to explore cell-based therapies, given that the surface expression of TIM-1 allows the isolation of Bregs, which could be expanded ex vivo and re-infused to patients with autoimmune disorders such as SSc, with the aim of replacing defective tolerance mechanisms.

## Additional file

Additional file 1: Figure S1. Representative dot-plots and column graphs showing the percentage of IL-10^+^ TIM-1^+^ in transitional, naïve, and memory B cell subpopulations from healthy donors, left unstimulated or activated with an anti-BCR antibody (*αBCR*) and CpG for 48 hours (n = 4). **P* < 0.05 (PDF 583 kb)

## Abbreviations

BCR: B-cell receptor
Bregs: regulatory B cells
CFSE: 5, 6-carboxylfluorescein diacetate succinimidyl ester
dcSSc: diffuse cutaneous systemic sclerosis
DLCO: Diffusing capacity for carbon monoxide
EAE: experimental autoimmune encephalomyelitis
ELISA: enzyme-linked immunosorbent assay
FMO: fluorescence minus one
IFN: interferon
IL: interleukin
lcSSc: limited cutaneous systemic sclerosis
PMA: phorbol 12-myristate 13-acetate
SSc: systemic sclerosis
Th: helper T cells
TIM-1: T cell Ig and mucin domain protein 1
TLR9: Toll-like receptor 9, Tregs, regulatory T cells
