# Supplementary figures and images for: TIM-1 defines a human regulatory B cell population that is altered in frequency and function in systemic sclerosis patients
Source: Arthritis Res Ther. 2017 Jan 19;19:8. doi: 10.1186/s13075-016-1213-9 (PMC5248463; doi:10.1186/s13075-016-1213-9)

# Unstimulated

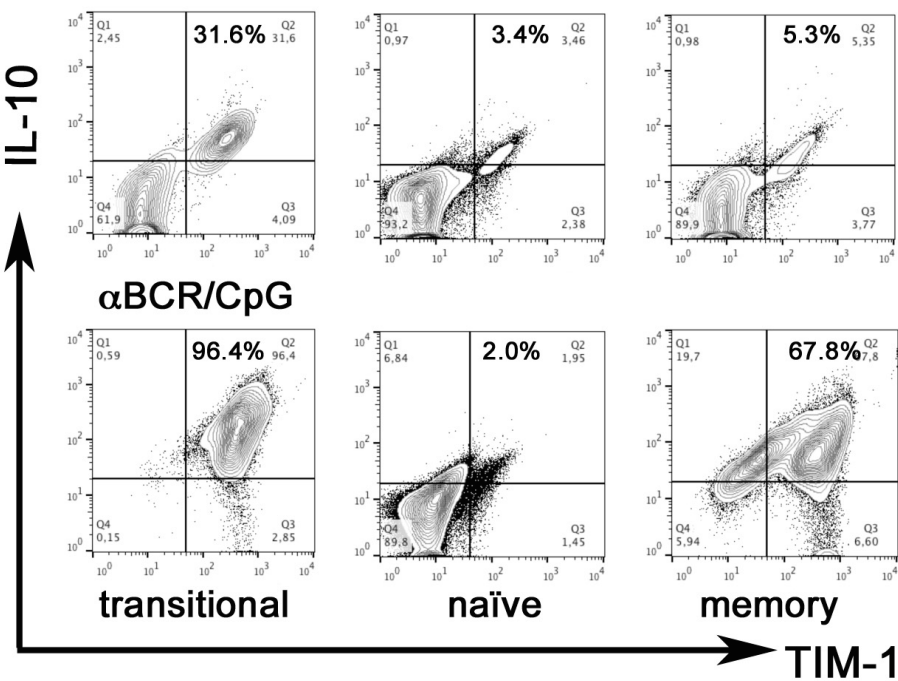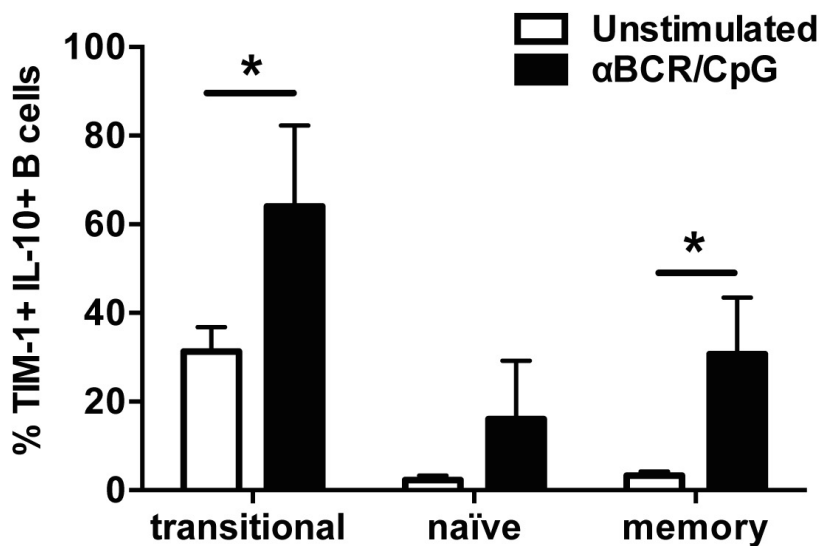

Supplement: Additional file 1: Figure S1. — Representative dot-plots and column graphs showing the percentage of IL-10+ TIM-1+ in transitional, naïve, and memory B cell subpopulations from healthy donors, left unstimulated or activated with an anti-BCR antibody (αBCR) and CpG for 48 hours (n = 4). *P < 0.05 (PDF 583 kb) [file 13075_2016_1213_MOESM1_ESM.pdf]
